# Teaching Analogical Reasoning With Co-speech Gesture Shows Children Where to Look, but Only Boosts Learning for Some

**DOI:** 10.3389/fpsyg.2020.575628

**Published:** 2020-09-23

**Authors:** Katharine F. Guarino, Elizabeth M. Wakefield

**Affiliations:** Department of Psychology, Loyola University Chicago, Chicago, IL, United States

**Keywords:** gesture, learning, visual attention, eye tracking, analogical reasoning

## Abstract

In general, we know that gesture accompanying spoken instruction can help children learn. The present study was conducted to better understand *how* gesture can support children’s comprehension of spoken instruction and whether the benefit of teaching though speech and gesture over spoken instruction alone depends on differences in cognitive profile – prior knowledge children have that is related to a to-be-learned concept. To answer this question, we explored the impact of gesture instruction on children’s analogical reasoning ability. Children between the ages of 4 and 11 years solved scene analogy problems before and after speech alone or speech and gesture instruction while their visual attention was monitored. Our behavioral results suggest a marginal benefit of gesture instruction over speech alone, but only 5-year-old children showed a distinct advantage from speech + gesture instruction when solving the post-instruction trial, suggesting that at this age, children have the cognitive profile in place to utilize the added support of gesture. Furthermore, while speech + gesture instruction facilitated effective visual attention during instruction, directing attention away from featural matches and toward relational information was pivotal for younger children’s success post instruction. We consider how these results contribute to the gesture-for-learning literature and consider how the nuanced impact of gesture is informative for educators teaching tasks of analogy in the classroom.

## Introduction

Gestures – movements of the hands that are naturally used in conversation and express ideas through their form and movement trajectory – help children learn. This has been found across domains, including mathematics (Singer and Goldin-Meadow, 2005; Cook et al., 2013), symmetry (Valenzeno et al., 2003), conservation (Church et al., 2004; Ping and Goldin-Meadow, 2008), and word learning (Wakefield et al., 2018a). And while this function of gesture is well-established, the mechanism by which gesture supports children’s learning, and how individual differences between children impacts the effectiveness of incorporating gesture into instruction, are not fully understood.

One way gesture is thought to help children learn is by grounding and disambiguating the meaning of spoken instruction (e.g., Alibali and Nathan, 2007; Ping and Goldin-Meadow, 2008). When learning a new concept, children may struggle to understand the meaning of spoken instruction and fail to see connections between a teacher’s speech and their use of supportive materials like equations, figures, or diagrams. Gestures facilitate connections between spoken language and these physical supports by directing attention to key components of a problem being taught or providing a visual depiction of an abstract concept through hand shape or movement trajectory (e.g., McNeill, 1992; Altmann and Kamide, 1999; Huettig et al., 2011; Wakefield et al., 2018b). For example, when being taught the concept of mathematical equivalence – the idea that two sides of an equation are equal to one another (e.g., 2 + 5 + 8 = _ + 8) – eye tracking results show that children follow along with spoken instruction more effectively if it is accompanied by gesture than if the concept is explained through speech alone. Importantly, children’s ability to follow along with spoken instruction when it was accompanied by gesture predicted their ability to correctly solve mathematical equivalence problems beyond instruction (Wakefield et al., 2018b).

However, incorporating gesture may not support all children’s understanding of spoken instruction to the same extent. Although prior work suggests that gesture supports children’s learning, there are nuances to when gesture is beneficial: Children’s pre-existing knowledge related to a domain – which we will refer to as their *cognitive profile* – can impact whether they learn from gesture instruction. For example, Wakefield and James (2015) taught children the concept of a palindrome (i.e., a word that reads the same forward and backward) through speech-alone or speech + gesture instruction. They considered whether the impact of gesture was affected by children’s relevant cognitive profile – in this case, their phonological ability, as the task relied heavily on understanding how sounds in words fit together. Children with high phonological ability benefitted more from speech + gesture instruction than speech-alone instruction, but children with low phonological ability did not show this advantage, suggesting that children need some degree of pre-existing knowledge within the domain to utilize gesture. In this case, the authors argued that gesture could not clarify spoken instruction unless children had a certain level of phonological awareness.

Although not considered by Wakefield and James, there may also be a developmental point when children are on the brink of understanding a concept and have a sufficiently developed cognitive profile that they need just a small boost from instruction to master a concept. In this case, incorporating gesture into instruction might not be any more powerful than spoken instruction alone. There may be a ‘sweet spot’ where children have enough foundational knowledge and cognitive abilities related to a concept that gesture can clarify spoken instruction and boost their learning, while children far below or above this developmental point do not show an advantage when learning through gesture.

In the present study, to better understand how gesture can support children’s understanding of spoken instruction and whether the benefit of teaching though speech and gesture over spoken instruction alone depends on differences in cognitive profile, we explore the impact of gesture in analogical reasoning. Analogical reasoning is the ability to identify underlying schematic or relational structure shared between representations. In its mature form, it is a powerful cognitive mechanism that contributes to a range of skills unique to humans (for review see Gentner and Smith, 2013). For the purpose of the present study, analogical reasoning is a useful testbed because it is a domain that requires disambiguating complex verbal information, and because the relevant cognitive profile for solving analogies shows protracted development across early childhood (e.g., Richland et al., 2006; Thibaut et al., 2010; Thibaut and French, 2016; Starr et al., 2018).

One of the predominant types of analogy task used to assess the development of children’s analogical reasoning ability are scene analogies, in which children are asked to examine two scenes (e.g., a source and target scene) which contain both relational similarities and featural similarities. When prompted to solve a scene analogy, children are asked to identify an item in the target scene that corresponds *relationally* to a prompted item in the source scene. However, children often choose an item that corresponds *featurally* to the prompted item instead of the relationally similar item. This type of ‘featural match’ is one item in a target scene that is not incorporated in the relation of focus, but has great surface similarity to the prompted item in a source scene (Richland et al., 2006). For example, a source scene might show a boy chasing a girl (relation of *chasing*), with the boy prompted. The corresponding target scene would contain a dog chasing a cat (relation of *chasing*) and a second boy (the featural match). Here, the dog would be the correct relational choice, and the second boy would be the incorrect featural match. Young children find it difficult to disengage from the featural match (i.e., another boy that is similar in appearance to the prompted boy) in favor of a relational match (i.e., the other thing that is chasing). This focus on surface features, or perceptual similarities, rather than relational information is a common pitfall for children (Gentner, 1988) that they may not fully overcome until they are 9–11 years of age (Richland et al., 2006).

Because incorporating gesture in instruction can direct children’s visual attention effectively to key components of a problem in other domains, such as mathematics instruction (Wakefield et al., 2018b), gesture should also be able to facilitate effective visual attention in problems of analogy. Gesture should be able to clearly indicate, and disambiguate, which items a teacher is referring to when providing spoken instruction, so that children are focused on items and relations relevant for successful solving and do not attend to irrelevant items. When considering the previous example of a scene analogy, a teacher is likely to align the important relations through speech, stating that the boy is chasing the girl, and the dog is chasing the cat. In theory, this type of statement, which highlights structural similarities between contexts, should orient children’s attention to the items involved in the relation of chasing, and, thereby, facilitate an analogical comparison (e.g., Gentner, 1983, 2010; Markman and Gentner, 1993; Namy and Gentner, 2002). However, when a featural match is present, this spoken instruction by itself may leave some ambiguity in terms of *which* boy is being discussed. Children may focus their attention on one or both boys (i.e., the boy in the chasing relation and the featural match) and miss the important connections being drawn between the relations in the source and target scenes. Indeed, we know from eye tracking studies that children who incorrectly solve analogical reasoning problems tend to focus their visual attention on the featural match, and ignore relational information (Thibaut and French, 2016; Glady et al., 2017; Starr et al., 2018; Guarino et al., 2019). Instruction that incorporates gesture may help young children understand which boy is relevant to the task and direct their attention away from irrelevant featural matches.

But will gesture instruction provide the same boost to all children who struggle to solve analogical reasoning problems? The determining factor may be a child’s cognitive profile relevant to analogical reasoning ability, comprised of effective inhibitory control and working memory. Inhibitory control allows an individual to inhibit more salient, featural match responses, and select a less salient, but correct, relational match (e.g., Viskontas et al., 2004; Richland et al., 2006). Working memory allows an individual to simultaneously process multiple contexts and pieces of information present in an analogy (e.g., Gick and Holyoak, 1980; Halford, 1993; Simms et al., 2018). Due to the protracted development of these cognitive capacities, analogical reasoning similarly develops gradually over time, with initial stages presenting in children as young as 3–5 years old and maturing into adolescence (e.g., Alexander et al., 1987; Goswami and Brown, 1989; Rattermann and Gentner, 1998). In the case of a scene analogy, Richland et al. (2006) find that children have difficulty ignoring featural matches in favor of relational matches until they are 9–11-years-old, with children showing an increase in successful problem solving between the ages of 3 and 11, as children’s cognitive profiles develop.

With this protracted development of cognitive profile in mind, we might expect differences in the effectiveness of gesture instruction. For very young children their inhibitory control and working memory may be so limited that they may not be able to capitalize on gesture’s ability to index spoken instruction to referents in a scene analogy, and therefore, gesture may not be helpful for disambiguating complex verbal instruction. However, for slightly older children, we may find that gesture provides the exact boost they need: They may have the cognitive profile in place to benefit from instruction, and gesture may give them an extra boost by literally pointing them in the right direction to help them make sense of spoken instruction. For even older children with high inhibitory control and working memory capacity, who typically demonstrate near-adult like ability on problems of analogy, receiving spoken instruction, even without gesture, may be enough support for understanding the structure of analogies.

### Present Study

We test these predictions in the present study. To do this, we compare how children across a wide age range (4–11-year-olds) solve scene analogy problems before or after speech alone or speech and gesture instruction while monitoring their visual attention with eye tracking. Using a wide age range will allow us to understand how cognitive profile contributes to the effectiveness of gesture instruction. Using eye tracking will allow us to understand how gesture aids in disambiguation of spoken instruction meant to refer to an item within a relation, that could instead be linked to a featural match. Through this approach, we will address three questions: (1) Do children benefit differently from speech alone versus speech and gesture instruction on analogical reasoning based on their age (as a proxy for cognitive profile)? (2) Can we find evidence that gesture instruction helps disambiguate spoken instruction, and does this depend on age? (3) Do looking patterns associated with type of instruction impact whether children at different ages learn from instruction? Results will add to our general understanding of the mechanisms by which children learn and explore the nuances of when gesture may or may not help beyond spoken instruction. And by focusing on analogical reasoning, we also explore the utility of gesture instruction in a domain that is important for academic success and has been understudied in the gesture-for-learning literature.

## Materials and Methods

### Participants

Children between the ages of 4 and 11 years old (*N* = 323; 159 females) participated in the present study during a visit to a science museum^1^. Children were randomly assigned to one of two conditions (*n*_speech–alone_ = 160; *n*_speech+gesture_ = 163), with a target of ∼20 children per age group in each condition. An additional 62 children participated in the study but were excluded from analyses for eye tracker malfunction (*n* = 20), parental involvement (*n* = 7), language barrier (*n* = 2), lack of response from participant (*n* = 7), poor eye tracking (*n* = 3), and experimenter error (*n* = 23). Two participants decided they did not want to continue before being assigned a condition. Informed consent was obtained from a parent or guardian of each participant, and verbal assent was obtained from children. Children participated individually in one 3–5 min experimental session and received stickers as compensation.

### Materials

#### Warm-Up Examples

Children were shown two scenes depicting relations occurring between items (see Appendix A for items). For example, a scene showed one animal (e.g., elephant) reading to another animal (e.g., rabbit), and another scene showed an animal (e.g., duck) on top of another animal (e.g., cow). Instruction was provided that highlighted the relation of interest (i.e., *patterns* of ‘reading’ and ‘on top of’). These trials served to familiarize children with our use of the term *pattern* and how items can be *relationally associated*.

#### Pre- and Post-instruction Stimuli

Two scene analogy problems (see Appendix A for items) were selected from a data set created by Guarino et al. (2019), that were based on the structure used by Richland et al. (2006). Scene analogies have been used in a number of other studies assessing the development of children’s analogical reasoning ability (e.g., Morrison et al., 2004; Richland et al., 2006, 2010; Gordon and Moser, 2007; Krawczyk et al., 2010; Morsanyi and Holyoak, 2010; Glady et al., 2016; Simms et al., 2018). Previous work has found that children as young as 3–4 years old can successfully solve scene analogy problems when there is not a featural match present just over half of the time (Richland et al., 2006). And by age 9–11 children are fairly proficient at solving scene analogies, even when featural matches are present (Richland et al., 2006). Therefore, this analogy format is particularly useful for assessing analogical reasoning ability across the age range utilized in the present study because it encompasses the entire developmental trajectory of this ability.

**FIGURE 1:**
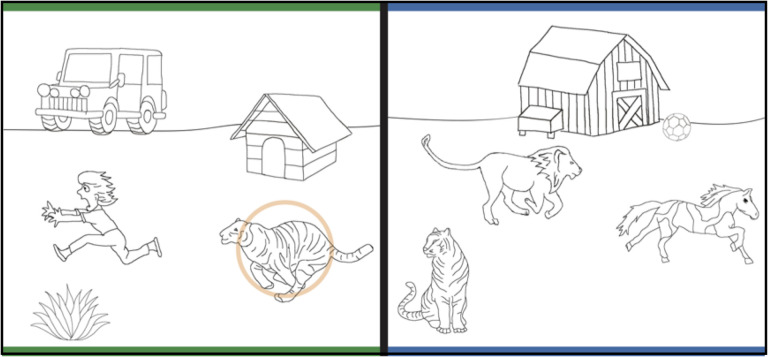
Example trial of a chasing relation stimulus.

Each problem included a pair of scenes, a source scene on the left, and a target scene on the right. Scenes depicted the relation *chasing* occurring between items (i.e., animals or people; Figure 1). Source scenes contained five items: the two items within the relation of chasing, and three additional items (i.e., neutral inanimate objects that were not involved in the relation of chasing). One of the items within the source scene relation was circled. Target scenes also contained five items: the two items within the relation, two additional items, and a featural match. The *featural match* was similar to the circled source-scene item and centrally located, increasing the likelihood that the item would draw participants’ attention.

Figure 1 shows an example of a chasing *source* and *target* scene. The source scene on the left shows a tiger chasing a woman (items within the *chasing* relation), and a dog-house, jeep and plant (neutral items). The corresponding target scene on the right shows a lion chasing a horse (items within the *chasing* relation), a barn and soccer ball (neutral items), and a tiger (a featural match item that is superficially similar to the prompted tiger in the source scene).

The directionality of relations within a pair of scenes was reversed to avoid children making choices based on spatial location alone. For example, in Figure 1, the direction of chasing is right to left in the source scene (the tiger on the right is chasing the woman on the left), whereas the direction of chasing is left to right in the target scene (the lion on the left is chasing the horse on the right). Children were presented with printed copies of scene analogies. Stimuli were bound in a binder, with one pair of scenes presented at a time.

#### Instruction Stimuli

Similar to pre- and post-instruction trials, printed instruction stimuli included two scenes in which a chasing relation was depicted in both scenes, and a featural match was located in the target scene (see Appendix A for items). Unlike pre- and post-instruction trials, no item was circled in the instruction stimuli.

#### Eye Tracker

Eye tracking data were collected via corneal reflection using a Tobii Pro Glasses 2. Tobii software was used to perform a 1-point calibration procedure. This step was followed by the collection and integration of gaze data using Tobii Pro Lab (Tobii Technology, Sweden). Data were extracted on the level of individual fixations as defined by Tobii Pro Lab software—an algorithm that determined if two points of gaze data are within a preset minimum distance from one another for a minimum of 100 ms, allowing for the exclusion of eye position information during saccades. After extraction, fixations were manually mapped by research assistants. Individual fixations were classified as either oriented toward one of the items of interest within the scenes (e.g., to the item chasing in the source scene, to the item being chased in the source scene, to the featural match, etc.), other areas around the items within the scenes, or the space surrounding the scenes. Research assistants assigned each fixation to an area of interest (AOI), based on its location (e.g., if a fixation was located on or within the immediate area surrounding the featural match, it was manually mapped as a featural match fixation). For more details about manual mapping, see Appendix B.

### Procedure

Children participated individually at a table in a corner of the museum floor. Children were told they were going to play a picture game while wearing eye tracking glasses. After a brief explanation that the purpose of the glasses is to ‘help us see what you see,’ an experimenter fitted them with the glasses. Children were seated approximately 40 cm in front of the printed stimuli next to an experimenter. The printed stimuli were displayed in a binder mounted on an easel. This allowed the experimenter to quickly flip between stimuli and gesture to the stimuli during instruction trials if a child was assigned to the speech + gesture condition. It also ensured proper eye tracking – children could see the stimuli directly in front of them, and did not have to look down toward the table, which would have disrupted our ability to capture their visual attention via the eye glasses. Children’s position was calibrated and adjusted if necessary, and they were asked to remain as still as possible during the rest of the game while eye tracking data were collected.

First, the experimenter explained the relational pattern in the two warm-up trials, meant to help promote relational thinking (see section “Materials and Methods” for details). Next, children completed one pre-instruction trial. After orienting children to the two scenes presented simultaneously (e.g., one side has blue edges and one has green edges), children were asked, “*Which thing in the picture with the blue edges is in the same part of the pattern as the circled thing in the picture with the green edges?*” An item in the source scene (e.g., in Figure 1, green edges) was circled and had a corresponding relational item and featural match in the target scene (e.g., in Figure 1, blue edges). All stimuli used in this task can be seen in Appendix A and additional details about the stimuli can be found in the “Materials and Methods” section. The task was self-paced, but if no response was given after a few seconds, the children were re-prompted by the experimenter.

Following the pre-instruction trial children were asked to pay attention to two instruction trials to learn about the pattern in the pictures. Children were randomly assigned to receive *speech-alone* instruction or *speech + gesture* instruction provided by the experimenter. In her instruction, the experimenter described chasing relations and similarities between items from a source and target scene, displayed in front of the child. For example, in a scene analogy problem with a boy chasing a girl in a source scene and a dog chasing a cat in a target scene with a featurally matched boy present, the experimenter said, “*See*, *the boy is chasing the girl, and the dog is chasing the cat. This means the boy is in the same part of the pattern as the dog because they are both chasing, and the girl is in the same part of the pattern as the cat because they are both being chased.*” The ambiguity of this instruction occurs when the boy in the source scene is referenced, because there is also a featurally similar boy in the target scene (i.e., the featural match). When the boy is mentioned in speech it may be difficult for children to reconcile *which* boy is being discussed: the one in the relation of chasing or the featural match. This confusion or ambiguity could contribute to difficulty identifying the relational structure in an analogy problem.

**FIGURE 2:**
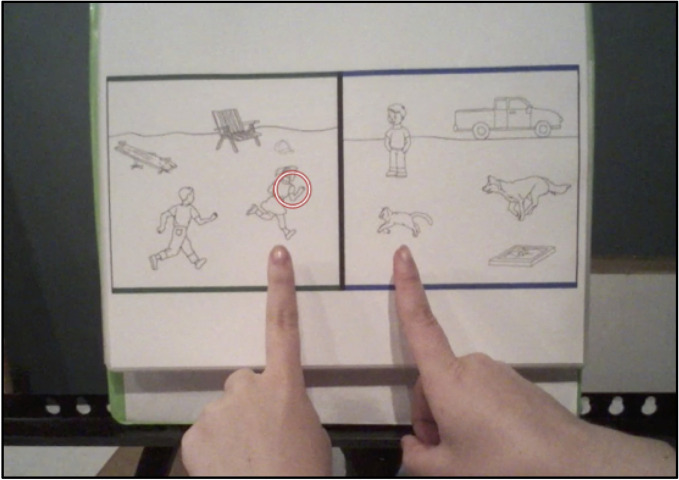
Example of children’s view during a speech + gesture training trial. The red circle shows where the child was focusing his or her visual attention at this moment of instruction.

In the speech + gesture condition, the experimenter provided the same spoken instruction, accompanied by gestures that emphasized items and relations. In the example above, when the experimenter said ‘*The boy is chasing the girl*,’ a sweeping movement of the index finger traced a path from the boy to the girl, highlighting the chasing relation. The same sweeping gesture was used when the experimenter said ‘… *and the dog was chasing the cat.*’ Then, deictic gestures – pointing gestures used to indicate objects or locations – were used to simultaneously reference the items that were in the same parts of the relations. Items were indicated by a pointed index finger on each hand. When the experimenter said, ‘*This means the boy is in the same part of the pattern as the dog because they are both chasing*,’ simultaneous deictic gestures pointed to the boy and the dog. Similarly, when the experimenter said, ‘…*and the girl is in the same part of the pattern as the cat because they are both being chased*,’ simultaneous deictic gestures pointed to the girl and the cat (see Figure 2 for an example of children’s view during training).

Finally, a post-instruction trial was administered after children viewed the instructional trials, with an identical prompt and procedure as used during the pre-instruction trial.

### Measures of Visual Attention

#### Measure of Attention During Pre- and Post-instruction Trials

Visual attention during pre- and post-instruction trials was quantified by generating areas of interest that represent different portions of the participant’s field of view using Tobii Pro Lab. There were 11 AOIs in total (see Appendix B). The AOIs encompassed regions within the scene pairs and areas in the field of view that were outside of the scene analogy. This included an AOI for each of the items in the scenes (items in chasing relations, featural match, and neutral items), AOIs for when the participant fixated on the experimenter, on the experimenter’s gesture, and on their own hands, and an AOI for looking elsewhere in the museum. Proportion of time spent looking to each AOI was then calculated by dividing the time looking to an AOI during a trial by the total time looking during a trial. For the sake of the present analyses, we focused on the AOI representing the featural match. Children’s ability to avoid featural matches is one of the key issues children overcome as they develop successful analogical reasoning. By assessing visual attention to the featural match we can assess whether gesture is more effective than speech alone for driving attention away from irrelevant featural components.

#### Measures of Attention During Instruction

Attention during instruction was quantified in two ways: (1) children’s ability to synchronize their visual attention with spoken instruction and (2) ‘check-ins’ with the featural match during ambiguous spoken instruction.

##### Following score

Because previous work suggests that gesture can help children follow along with spoken instruction and that this is predictive of learning (Wakefield et al., 2018b), we calculated a ‘following score’ for each instruction trial. Following scores were calculated by creating four time segments in which different relational comparisons were made by the experimenter and assessing whether children looked to AOIs highlighted in speech during each segment (i.e., during a given segment, children received a score of ‘1’ if they looked to the relevant AOIs as they were labeled in speech and a ‘0’ if they did not). Children could receive a score of 0 to 4 on each training trial, and scores were averaged across the two training trials to generate an overall following score for each child. The average following score was used in analyses.

##### Check-in score

Check-ins with the featural match are instances when the item that is perceptually similar to the featural match is referenced in speech and simultaneously fixated on by the child. In each instruction trial, there were two time segments during which a check-in could occur. For example, in the instruction trial depicting a boy chasing a girl in the source scene and a featural match boy in the target scene, the two relevant time segments occur when the experimenter said ‘*The boy is chasing the girl’* and ‘*The boy is in the same part of the pattern as the dog because they are both chasing.’* For each segment, a child would receive a score of 1 if they looked to the featural match boy in the target scene rather than the boy in the source scene. Children would receive a score of 2 for a given instruction trial if they looked at the featural match boy during both time segments in which the boy in the relation was mentioned. Thus, whereas a score of 4 is possible for following score, a score of 2 is possible for check-in score. Check-in scores from the two instruction trials were averaged to generate an overall check-in score for each child. The average ‘check in’ score was used in analyses.

## Results

All analyses were conducted using R Studio (version 1.1.456), supported by R version 3.6.0. Analyses relied on the *stats* package, which allows for ANOVA and regression modeling (R Core Team, 2017). When running binomial generalized linear regression models assessing the impact of condition on accuracy or choice of the featural match at pre- and post-instruction, the speech-alone condition was set as the baseline condition and compared against the speech + gesture condition. For analyses of visual attention, which did not use a binomial outcome, generalized linear regression models were used. Again, speech-alone was set as the reference level for these analyses.

Before addressing our main questions of interest, we wanted to establish (1) that there were no significant performance differences pre-instruction between children who had been randomly assigned to the speech-alone versus speech + gesture condition – we found that there were not: Both across all children and within age groups, there were no condition differences between pre-instruction accuracy or choice of the featural match (all *ps* > 0.1), and (2) that age could serve as a proxy for cognitive profile. To do this, we asked whether children’s ability to solve analogical reasoning problems could be predicted by age and visual attention before instruction. We reasoned that previous work has shown that as children’s inhibitory control and working memory improve, they are more likely to succeed on analogical reasoning problems (e.g., Doumas et al., 2018; Simms et al., 2018), and that children with lower inhibitory control look more to the featural match when solving scene analogy problems (Guarino et al., under revision), thus, finding that age was predictive of these measures would suggest that age can serve as a proxy for cognitive profile.

**FIGURE 3:**
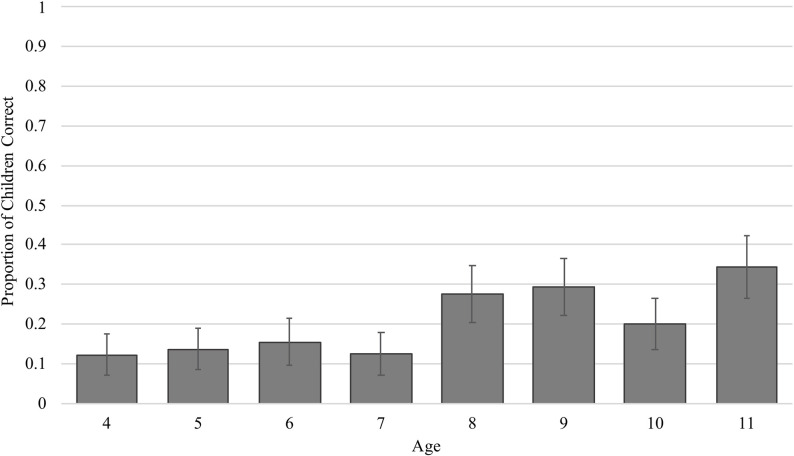
Proportion of children within each age correct on the pre-instruction trial.

While only 20% of children correctly answered the pre-instruction trial, there was a main effect of age when predicting accuracy, such that older children were more likely to answer the problem correctly than younger children (Figure 3, β = 0.18, *SE* = 0.06, *t* = 2.89, *p* = 0.004), replicating previous work (e.g., Richland et al., 2006). And, as with previous studies using scene analogy problems, we found that the most common error children made was to choose the featural match – 64% of children made this type of error. In terms of visual attention, we assessed whether children’s proportion looking to the featural match before instruction predicted their performance, as this is a key looking pattern associated with making featural errors (e.g., Thibaut et al., 2010; Thibaut and French, 2016; Guarino et al., 2019). On average, children who correctly answered the pre-instruction trial allocated less of their attention to the featural match (*M* = 0.12, *SD* = 0.08) than children who made featural errors (*M* = 0.14, *SD* = 0.08). Models predicting accuracy by visual attention to the featural match showed that proportion looking to the featural match was negatively related with accuracy (β = −0.00, *SE* = 0.00, *t* = −2.22, *p* = 0.026) and positively related with featural errors (β = 0.00, *SE* = 0.00, *t* = 3.51, *p* < 0.001). In sum, these results replicate previous work finding that prior to instruction, children who are older and attend less to the featural match more successfully solve a scene analogy problem, and provide support for considering age as a proxy for cognitive profile.

### Impact of Age and Instruction on Children’s Analogical Reasoning Ability

**FIGURE 4:**
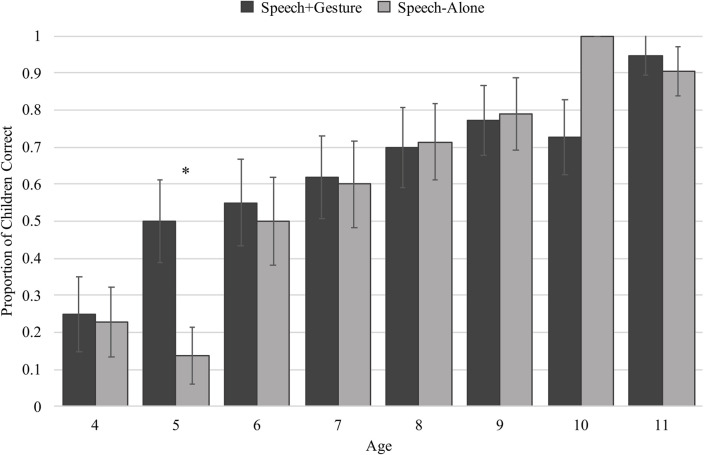
Proportion of children within each age correct on the post-instructional trial separated by condition. ^∗^indicates significance at *p* > 0.05

To understand how speech-alone versus speech + gesture instruction affected children’s performance on the post-instruction trial, we limited the remainder of our analyses to children who incorrectly answered the pre-instruction trial (speech-alone: *n* = 124; speech + gesture: *n* = 133) – importantly, a similar number of children were excluded from both experimental groups. Our first main question was whether the impact of gesture instruction on children’s analogical reasoning is dependent on their cognitive profile (measured by age). Overall, more children in the speech + gesture condition correctly answered the post-instruction trial than children in the speech-alone condition (speech + gesture: 63% vs. speech-alone: 59%). But, from Figure 4 it is clear that performance is also dependent on age, and when considering performance binned by age, we see that the difference between conditions appears most pronounced for 5-year-olds. To determine whether these patterns were statistically significant, we constructed a generalized linear model with accuracy (0, 1) as the dependent measure, and age, condition (speech-alone, speech + gesture), and an interaction between age and condition as predictors of interest. In line with Figure 4, the model revealed a main effect of age, suggesting that older children performed better after instruction than younger children (β = 0.62, *SE* = 0.12, *t* = 5.35, *p* < 0.001), and a trending main effect of condition, suggesting that children improved marginally more after speech + gesture instruction than speech-alone instruction (β = 1.82, *SE* = 1.06, *t* = 1.72, *p* = 0.085). However, these results should be considered within the context of a marginal interaction between age and condition (β = −0.25, *SE* = 0.15, *t* = −1.69, *p* = 0.092), where *post hoc* analyses indicate that only 5-year-old children demonstrate a benefit for speech + gesture compared to speech-alone (β = 1.75, *SE* = 0.89, *t* = 1.97, *p* = 0.048), and for all other children, there was not an effect of condition (*p*s > 0.1). Although this interaction was only marginally significant, this is likely due to the consideration of such a wide age range, with most age groups showing a clear lack of difference in response to instruction condition. The presence of an interaction aligns with the *a priori* hypothesis that gesture may only boost learning beyond speech-alone instruction at certain ages. Given previous work within the analogical reasoning literature that shows 5-year-olds demonstrate greater difficulty with problems incorporating featural matches than older children (e.g., Richland et al., 2006; Simms et al., 2018), it makes sense that gesture would provide these children the most benefit.

### Gesture’s Effect on Visual Attention During Instruction

Gesture instruction has previously been shown to help children follow along with spoken instruction and facilitate performance on subsequent assessments (Wakefield et al., 2018b). To understand how visual attention might play a role in the marginal behavioral effects of gesture on children’s post-instruction performance, we next asked how condition and age influenced children’s visual attention during instruction. Here, we used two measures of visual attention: following score and featural match check-in score. Children’s following score is an index of whether they looked at relevant referents of the problem (i.e., items involved in the relation of chasing) when the referents were mentioned in spoken instruction. Children’s featural match check-in score is an index of whether children attended to the featural match when the instructor’s speech was meant to reference an item within a chasing relation, but was ambiguous. Without understanding the context of the analogy, children could associate the spoken referent with either an item within a relevant chasing relation (the item the instructor meant to reference) *or* the featural match to that item (an item that is irrelevant to the analogy). Attending to the featural match may disrupt a child’s ability to effectively learn from instruction because it detracts from children’s ability to process how the items within the two chasing relations are aligned.

**FIGURE 5:**
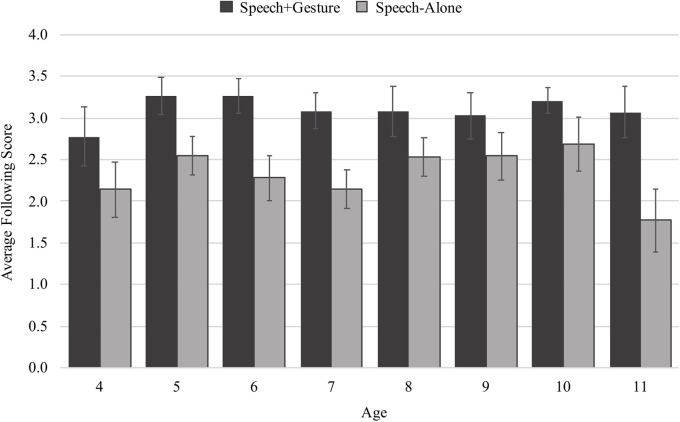
Average following scores split by age and condition.

On average, children followed along more successfully with spoken instruction if they were taught through speech + gesture (*M* = 3.08 out of a possible score of 4, *SD* = 1.10) than through speech alone (*M* = 2.20, *SD* = 1.06). Figure 5 shows following score separated by age and condition and suggests that gesture supports effective following along with instruction for all children. Using a generalized linear model with following score as the dependent measure and age, condition (speech-alone and speech + gesture), and an interaction between age and condition as the predictors of interest, we found a main effect of condition, confirming that speech + gesture instruction supported more effective following than speech-alone instruction (β = 1.51, *SE* = 0.26, *t* = 5.81, *p* < 0.001). We also found no main effect of age (β = 0.02, *SE* = 0.06, *t* = 0.28, *p* = 0.783) and no interaction between age and condition (β = 0.12, *SE* = 0.12, *t* = 0.25, *p* = 0.806) suggesting that gesture is a cue that can organize visual attention regardless of a child’s age.

Our second measure of visual attention during instruction was how children attended to the featural match, the key component of an analogy that draws children’s attention away from the relational information (e.g., Thibaut et al., 2010; Thibaut and French, 2016; Guarino et al., 2019). Specifically, we asked whether children attended to the featural match during the time intervals when the spoken instruction was ambiguous as to whether the instructor was referring to an item within a relation, or the featural match to that item outside of the relation (e.g., Which boy is being referred to: the boy in the relation of chasing or the featural match boy?). Because the lure of featural matches are at the root of young chilldren’s difficulties with problems of analogy, the most ambiguous portion of the instruction is when the item that is involved in the relation of chasing and perceptually similar to the featural match is discussed in speech.

**FIGURE 6:**
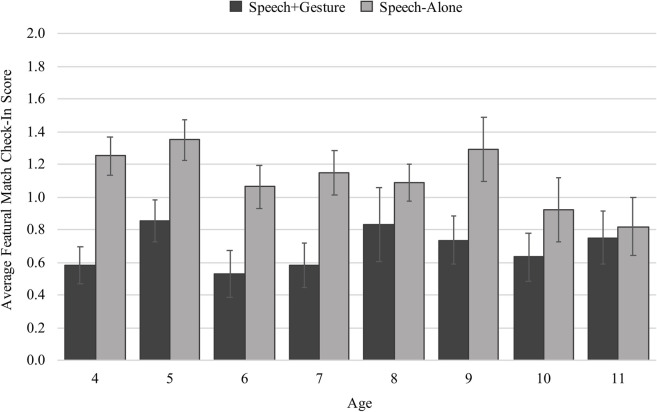
Average check-in scores split by age and condition.

On average, children checked-in more with the featural match if they were in the speech-alone condition (*M* = 1.29 out of a possible score of 2, *SD* = 0.48) than in the speech + gesture condition (*M* = 0.71, *SD* = 0.57). But again, the amount of difference between conditions seems to differ by age (Figure 6). Using a generalized linear model with check-in score as the dependent measure, and age, condition (speech-alone and speech + gesture), and an interaction between age and condition as the predictors, we found a main effect of condition, such that speech + gesture instruction facilitates fewer check-ins than speech-alone instruction (β = −1.77, *SE* = 0.47, *t* = −3.76, *p* < 0.001), and a main effect of age, such that older children check-in with the featural match less than younger children regardless of the type of instruction received (β = −0.11, *SE* = 0.05, *t* = −2.36, *p* = 0.019). These effects should be interpreted within the context of a trending interaction between condition and age (β = 0.00, *SE* = 0.06, *t* = 1.95, *p* = 0.052). *Post hoc* analyses indicate that this trending interaction results from a developmental shift between younger and older children (Table 1): Generally, older children are less likely to show a significant difference in check-in score across conditions, suggesting that they can make use of either speech-alone or speech + gesture instruction to avoid the featural match. In contrast, younger children’s visual attention is oriented away from the featural match more effectively by speech + gesture than the speech-alone instruction. This suggests that younger children use the added support of gesture to disambiguate speech and orient their attention away from featural matches.

**TABLE 1:** *Post hoc* analyses for testing condition effects predicting featural match check-ins.

**Age**	**Beta (*SE*)**	***p*-value**
4 year-olds	−1.33 (0.32)	<0.001
5 year-olds	−0.92 (0.35)	**0.012**
6 year-olds	−1.07 (0.38)	**0.008**
7 year-olds	−1.13 (0.37)	**0.005**
8 year-olds	−0.51 (0.44)	0.259
9 year-olds	−1.11 (0.47)	**0.024**
10 year-olds	−0.58 (0.46)	0.216
11 year-olds	−0.14 (0.46)	0.770

*Bolded *p* values indicate significant condition effects.*

In sum, the main effect of condition for following score suggests that gesture is effective for directing all children’s attention to the referents of spoken instruction. However, when considering the ambiguous portion of instruction, we see differences across age in the relative effectiveness of instruction. For older children, the alignment provided in spoken instruction, “*See, the boy is chasing the girl, and the dog is chasing the cat*” is enough context to recognize that when the instructor refers to the ‘*boy chasing the girl’* that the boy being referenced is the boy in the chasing relation, not the featural match that is outside of the relation: there is no added benefit of gesture for disambiguating speech. However, for the younger children, we see that gesture *does* have an effect. Children are less likely to look to the featural match when they receive speech and gesture instruction, compared to speech alone instruction. This suggests that gesture is helping disambiguate spoken instruction for these younger children.

### Impact of Visual Attention During Instruction on Children’s Analogical Reasoning

Having established that gesture does impact visual attention during instruction, whether this is for all children (following) or only children of particular ages (featural match check-in), we ask whether these patterns of visual attention can explain our behavioral results – that overall speech + gesture seems to marginally improve performance compared to speech-alone, but that this effect is driven by 5-year-old children, who show significantly better performance following speech + gesture instruction compared to speech-alone instruction.

To understand the relation between following along during instruction and performance on the post-instruction trial, we asked whether trial accuracy (0, 1) was predicted by following score. Age was not included in the model, as we found that it was not a relevant predictor of following. Our model revealed that following score was not predictive of accuracy (β = 0.07, *SE* = 0.06, *t* = 1.08, *p* = 0.280). This suggests that even though gesture helps children follow along with spoken instruction, this organization of visual attention does not contribute to its learning effects in the case of scene analogies.

To understand the relation between checking in with the featural match during instruction and performance on the post-instruction trial, we took into account our finding that, in general, younger children checked in less with the featural match when they received speech + gesture instruction than speech-alone instruction, but older children did not show this difference. This distinctly different pattern of results between younger and older children motivated the use of a median split by age (see Wakefield et al., 2017 for a similar approach): we constructed two models to ask whether check-ins during instruction were predictive of performance on the post-instruction trial for older (8–11 years) and younger (4–7 years) children separately. Here, we found that, whereas older children’s check-ins with the featural match did not significantly predict their accuracy at post-instruction (β = −0.12, *SE* = 0.18, *t* = −0.66, *p* = 0.512), younger children’s check-ins with the featural match *were* predictive of their performance on the post-instruction trial: check-ins were negatively related to successful problem solving (β = −0.45, *SE* = 0.18, *t* = −2.58, *p* = 0.009). This suggests that the ability of gesture instruction to direct attention away from the featural match and disambiguate the meaning of an instructor’s speech is the critical factor impacting analogical understanding for younger children.

## Discussion

The goals of the present study were to explore whether the impact of adding gesture to spoken instruction on analogical reasoning depends on children’s cognitive profile, and to use eye tracking to further understand how gesture might facilitate learning by disambiguating spoken instruction. Our behavioral results suggest a marginal benefit of gesture instruction over speech alone, but only 5-year-old children showed a distinct advantage from speech + gesture instruction when solving the post-instruction trial. This suggests that age – which we demonstrated was a good proxy for cognitive profile based on the relation between performance measures, visual attention, and age, in keeping with previous literature – does impact the utility of gesture for supporting analogical reasoning ability. To understand how disambiguation of speech may play a role in these results, we turned to eye tracking. We found evidence that gesture helps children follow along with spoken instruction, but that this was not predictive of successful problem solving post instruction. Rather, check-in score – visual attention toward the featural match at the point in instruction that was most ambiguous – was negatively predictive of post instruction success for younger children, but not for older children. This lends support to previous arguments that at the root of children’s struggle with analogical reasoning is an inability to ignore featural, or superficial, matches in favor of relational matches, and that looking to the featural match is associated with making these types of errors (e.g., Thibaut et al., 2010; Thibaut and French, 2016; Guarino et al., 2019). Although more work must be done to fully explore the impact of gesture instruction for analogical reasoning, these results suggest that one way gesture may help learning in this domain is through directing visual attention in a way that clarifies spoken instruction, but how much of a boost children get depends on their cognitive profile.

Our results suggest that in the case of analogical reasoning, gesture’s ability to disambiguate speech may be particularly useful for 5-year-old children who have the foundational cognitive abilities in place to benefit from gesture during instruction. Five-year-old children may be at a pivotal time in development of analogical reasoning ability: while they have a limited cognitive profile and immature analogical reasoning, their inhibitory control and working memory capacity are developed to the point that they can utilize the added support gesture provides. This finding that prior knowledge and ability impacts the utility of gesture corroborates other work in the gesture-for-learning literature. Children need some degree of prior knowledge within a domain that serves as a foundation that gesture instruction can build from (Wakefield and James, 2015; Congdon et al., 2018).

Importantly, our eye tracking data suggest what the added benefit of gesture might be: 5-year-old children showed an increased ability to follow along with instruction and less check-ins with the featural match when they learned through speech and gesture instruction versus speech alone instruction. Thus, the argument could be made that gesture is helping organize children’s visual attention in relation to spoken instruction and clarifying ambiguous instruction. But, only check-ins predicted success on the post instruction trial. Considering this in relation to previous work with eye tracking, this may seem puzzling. Wakefield et al. (2018b) found that following along with spoken instruction *did* predict subsequent performance in the case of mathematical equivalence. However, in their measure of following, spoken instruction was ambiguous; whereas in the present study, the general measure of following encompassed spoken instruction that was predominately not inherently difficult for children to decipher because the majority of items referenced in speech could only be associated with one unique item in a scene. In contrast, the speech during the featural match check-in measure *was* ambiguous, and is thus more analogous to the measure of following used by Wakefield et al. (2018b). In both of these cases, gesture is effective at clarifying parts of spoken instruction that are ambiguous, yet critical, for learning. Taken together, results from the current study and previous work suggest that gesture’s power to disambiguate spoken instruction is an important mechanism by which gesture shapes learning. And in the case of analogical reasoning, gesture can help children overcome one of the most challenging aspects of problem solving: clarifying for these children which items are in the relation of chasing and critical for solving the analogy, by helping them avoid the lure of a featural match.

While 5-year-olds may be in the developmental ‘sweet spot’ to benefit from gesture instruction, why does incorporating gesture not benefit all children equally? For all other children, those younger and older than 5 years, there was not a significant benefit of speech and gesture, compared to speech alone instruction, on post-instruction performance. It makes sense that older children (8–11-year-olds) demonstrated learning after both types of instruction: these children seemingly have all the necessary cognitive abilities and prior knowledge needed to utilize either type of instruction. Even though they struggled prior to instruction, their more developed inhibitory control and working memory allowed them to learn even from speech-alone instruction, and the addition of gesture is not necessary for learning the task. This is evidenced by the lack of difference between the number of check-ins with the featural match in the speech + gesture versus speech-alone conditions. Likely because they had the capacity to hold more information in working memory, they were able to consider the instructor’s alignment of the chasing relations and recognize which items were being referenced during instruction based on spoken instruction alone, and did not need gesture to organize their visual attention and help them make sense of instruction.

On the other end of the age range, the youngest children, 4-year-old children, may not have a sufficient cognitive profile in place to benefit more from gesture instruction than speech alone instruction. While gesture supports effective visual attention during instruction for these children, their inhibitory control and working memory may be too underdeveloped to extend their understanding beyond the moment, when the support of gesture is no longer immediately present. Thus, even though they looked to the featural match less in the gesture condition, they could not process the multiple relations mentioned in spoken instruction effectively.

Interestingly, 6- and 7-year-old children did not perform similarly to 5-year-old children or older children. While their visual attention was more effectively guided by a combination of speech and gesture instruction, as seen with their younger peers, they did not show the added benefit of gesture post instruction. The non-significant difference between conditions at post-instruction performance for these children may speak to their ability to disambiguate the instructions to some extent when only speech was provided. That is, these children may be able to disambiguate the instructions even with speech alone to a greater extent than 4- or 5-year-olds, but not as effectively as older children. And because they have slightly more mature cognitive profiles (i.e., more developed inhibitory control and working memory) than younger children, they may be better equipped to extend their understanding gained during instruction to post-instruction solving. Together, these results reflect that children’s cognitive profile makes a difference for whether gesture facilitates learning above and beyond speech alone instruction.

While this work makes strides toward understanding the nuances of gesture’s effects on learning, there are potential limitations that should be addressed. First, we suggest that age can serve as a proxy for a child’s cognitive profile without having independent measures of inhibitory control or working memory. Although collecting independent measures of inhibitory control and working memory would have been ideal, previous work using scene analogies has established that inhibitory control and working memory correlate with children’s age (5–11-years-old: Simms et al., 2018) and with their analogical reasoning ability over development (working memory: Simms et al., 2018; inhibitory control: Guarino et al., under revision), *and* that children’s visual attention is correlated with performance and inhibitory control (Guarino et al., under revision). Specifically, inhibitory control, measured using the Erikson Flanker task, is positively correlated with accuracy and attention to relationally similar items prior to instruction, and negatively correlated with choosing the featural match and attention to the featural match. Therefore, while it may be advantageous in future work to collect direct measures of children’s cognitive profile, here, we find the same relation between age, visual attention patterns, and analogical reasoning ability as has been documented in previous work. We are therefore confident that, motivated by previous work, age is associated with cognitive profile.

A second potential limitation is the length of our intervention, which consisted of one pre-instruction trial, two instruction trials, and one post-instruction trial. We designed the study based on previous gesture-for-learning literature showing children *can* benefit from a short intervention (Valenzeno et al., 2003; Church et al., 2004; Rowe et al., 2013). For example, Church et al. (2004) tested children’s knowledge of three types of Piagetian conservation (water, length, and number) using one question about each type of conservation before and after they watched one instructional video about conservation that either incorporated speech and representational gestures or speech alone. Similarly in the analogical reasoning literature, Gentner et al. (2016) tested how well children can analogically compare separate contexts after a short intervention. They first exposed children to one pair of model skyscrapers that varied in degree of alignment based on experimental condition, and then asked them build a structure as tall as possible that was ‘strong’ and repair a structure so it was ‘strong.’ Through successful comparison of the two model skyscrapers children could identify that a diagonal brace helps make a building ‘stronger.’ In the present study, we did find an effect of gesture instruction, above-and-beyond that of speech alone instruction, for children at a pivotal point in their analogical reasoning development. This suggests that once again, gesture can impact performance in a short period of time. However, it would be interesting to conduct future work lengthening the period of instruction, as this may allow children more opportunity to benefit from instruction, especially younger children who may need more examples to support their learning.

Finally, while not a limitation, the current work represents a starting, not an ending point, motivating additional questions to answer. For example, similar work using the test-bed of analogical reasoning should consider even younger children. The children in this study likely all had an underdeveloped, but nevertheless present, relevant cognitive profile to support the rudimentary stages of analogical reasoning (e.g., Davidson et al., 2006). Even 4-year-olds have been shown to have some degree of inhibitory control and working memory that allow them to make very simple comparisons – one of the basic building blocks for mature analogical reasoning (e.g., Davidson et al., 2006). To more fully understand the impact of gesture on children with little to no relevant cognitive skills, one could extend and adapt this task to incorporate 2- or 3-year-olds, given that some suggest children younger than 4-years-old have rudimentary relational reasoning capabilities (e.g., Goswami and Brown, 1989; Rattermann and Gentner, 1998; Ferry et al., 2015). The expectation would be that younger children, like 4-year-olds, would not benefit from gesture more than speech alone, and would strengthen the conclusions drawn from the present data.

Additionally, the impact of gesture is not only nuanced in terms of children’s current cognitive profile, but many other contextual or situational factors have been cited as playing a role in the effect on learning. For example, the advantage of speech + gesture compared to speech-alone instruction is not always evident in immediate measures at post-instruction, but rather in follow-up measures, from 24 hours (Cook et al., 2013) to 4 weeks (Congdon et al., 2017) after initial training. The one-trial post-instruction assessment may have limited the evaluation of learning.

In sum, the results of the present study extend our understanding of how gesture instruction impacts learning to the domain of analogical reasoning, while providing further insight into how gesture can help disambiguate spoken instruction and how individual differences in a child’s cognitive profile impacts the utility of gesture. These findings have important implications for designing teaching methods to support analogical reasoning, but also using gesture as a teaching tool more broadly. Because analogical reasoning shows such a protracted development, due to a slowly developing cognitive profile, it seems that only at certain points will gesture help children more than speech only instruction. Recognizing when this tool can be used could lead to faster growth in a skill that is at the root of a wide range of cognitive skills, such as innovation and creativity (for review see Halford, 1993). More broadly, this work speaks to one of the reasons *why* gesture helps learning, but also emphasizes that individual differences influence the impact gesture can have. Future work should continue to delve into the mechanisms by which gesture shapes learning and consider a child’s cognitive state as an important piece of this puzzle.

## Data Availability Statement

The data that support the findings of this study are openly available in Open Source Framework at https://doi.org/10.17605/OSF.IO/PAQ4S.

## Ethics Statement

The studies involving human participants were reviewed and approved by the Institutional Review Board of Loyola University Chicago. Written informed consent to participate in this study was provided by the participants’ legal guardian/next of kin.

## Author Contributions

KG and EW: conceptualization, formal analysis, project design and methodology, project administration and supervision, managing resources and software, validation of methodology, writing – original draft, and writing – review and editing. KG: data curation and investigation. EW: funding acquisition. Both authors contributed to the article and approved the submitted version.

## Conflict of Interest

The authors declare that the research was conducted in the absence of any commercial or financial relationships that could be construed as a potential conflict of interest.

## Footnotes

1 Although we did not collect demographic information from individuals, our sample was representative of the general profile of museum visitors. According to museum reports based on short surveys with museum visitors, visitors to the museum represent a number of different racial and ethnic backgrounds (70% White, 10% Hispanic, 6% African American, 6% Asian, 5% Other, <1% Native American, Native Hawaiian), and are also diverse in socioeconomic status, based on self- report measures of perceived socioeconomic status (13% lower or lower-middle class, 54% middle class, 33% upper middle or upper class) and parent or guardian’s highest level of formal education (1% < high school diploma, 18% high school diploma, 16% associates degree, 35% bachelor’s degree, 21% master’s degree, 7% Ph.D., or other terminal professional degree, 3% not reporting).
