# Supplementary material for: Teaching Analogical Reasoning With Co-speech Gesture Shows Children Where to Look, but Only Boosts Learning for Some
Source: Front Psychol. 2020 Sep 23;11:575628. doi: 10.3389/fpsyg.2020.575628 (PMC7538547; doi:10.3389/fpsyg.2020.575628)
Supplement: Supplementary file 1 [file Data_Sheet_1.pdf]

Appendix A: Stimuli

Appendix A includes all stimuli that were used during warm-up, pre-post instruction, and training trials. The order of pre-post instruction trials was counterbalanced across participants.

Warm-up Trial 1

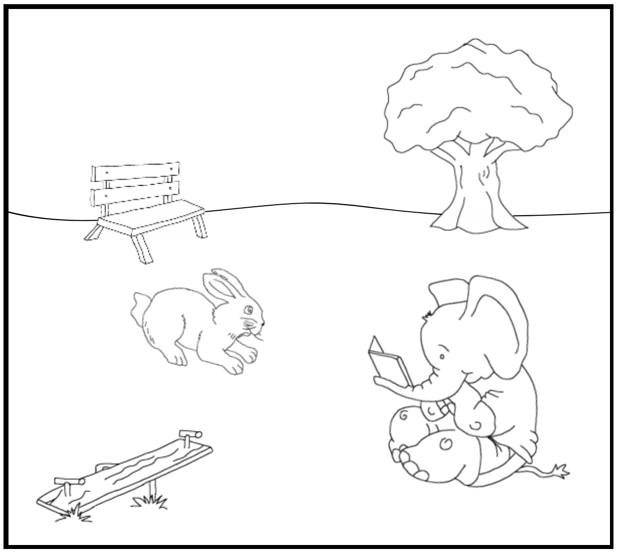

Warm-up Trial 2

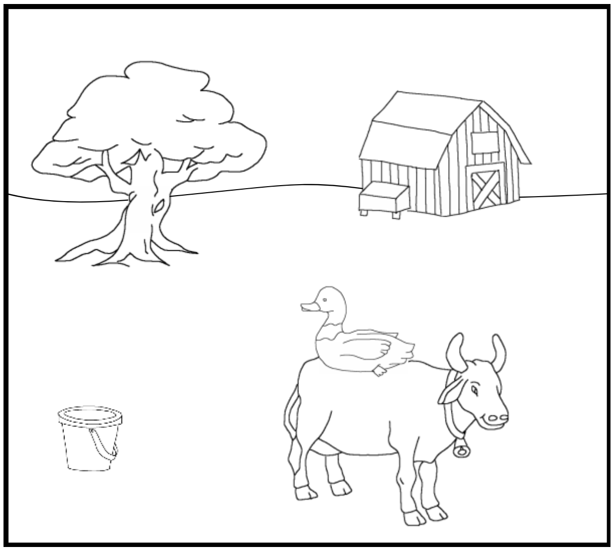

Pre/Post-Instruction Trials

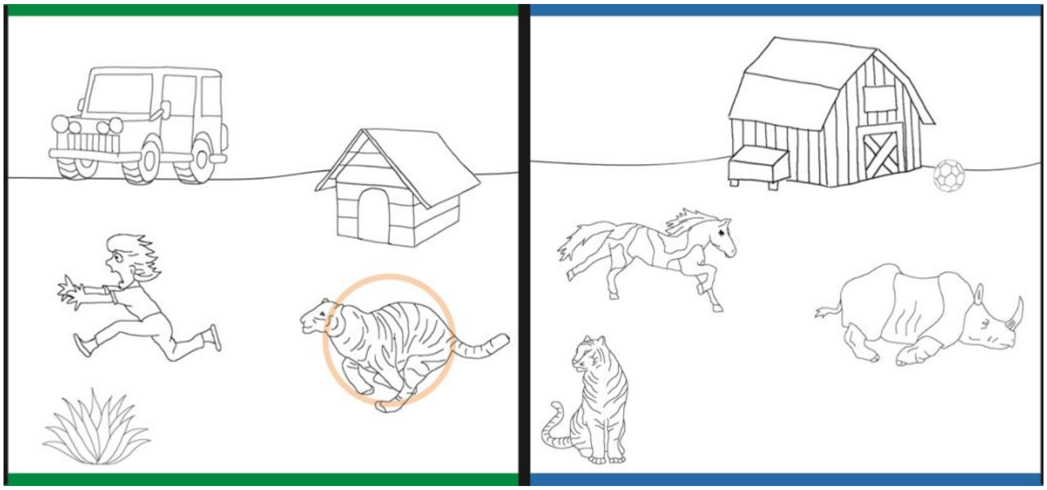

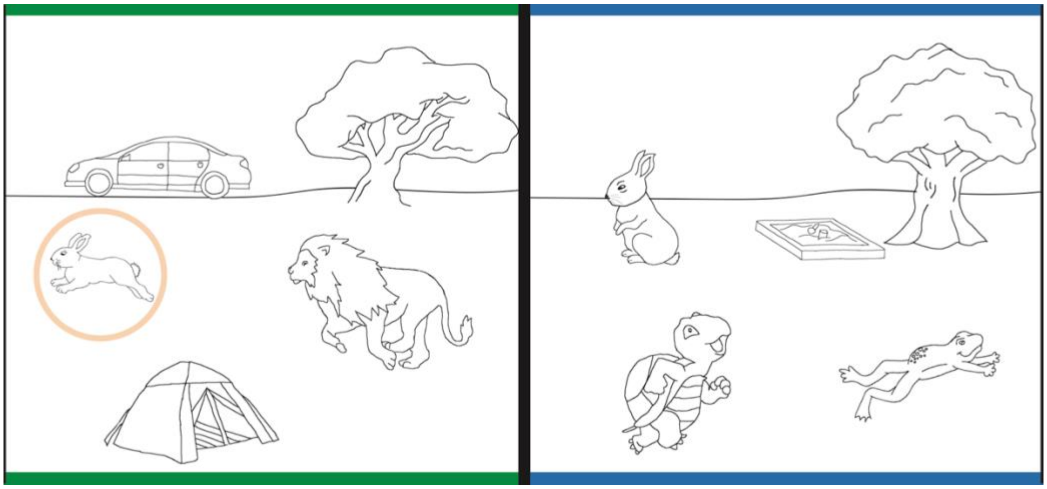

Instructional Trial 1

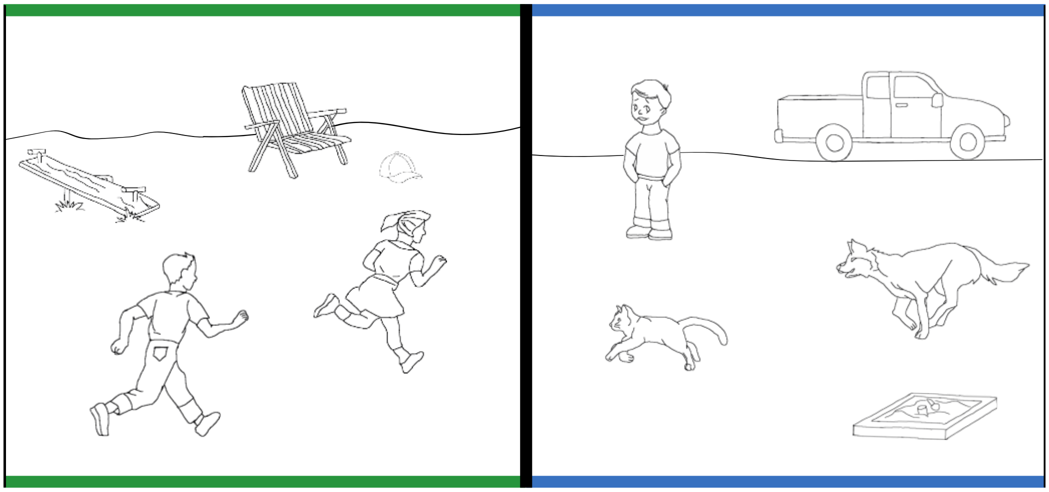

Instructional Trial 2

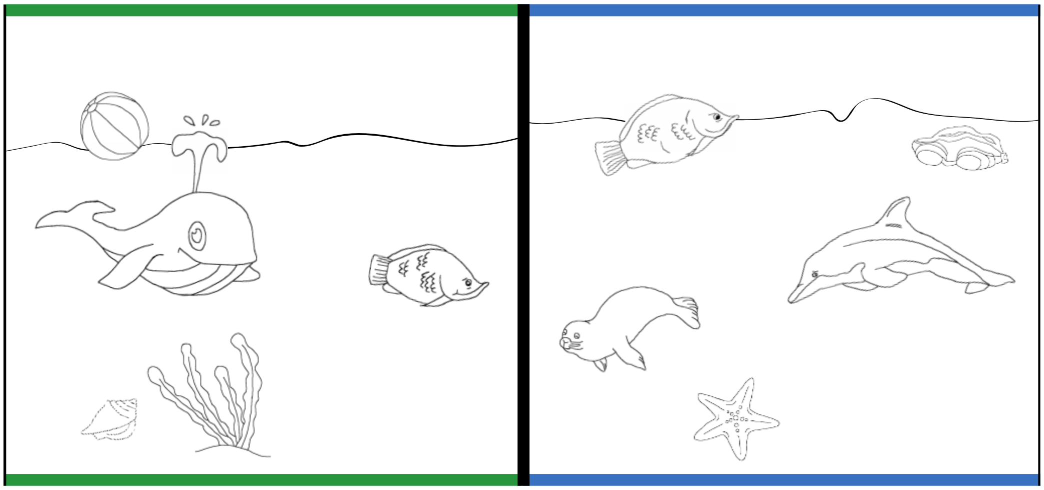

Appendix B: Areas of Interest (AOI)

Appendix B includes an illustration of one pre-post scene analogy trial with overlaid AOIs. The colored regions encompassing each item in the scenes represents the respective AOI for that item. Fixations were manually mapped by research assistants in Tobii Pro Lab (Tobii Technology, Sweden). This manual mapping was necessary because the field-of-view was dynamic, as children moved around while wearing the eye tracking glasses and thus the location and size of AOIs changed across time. Research assistants viewed the recording of participant's eye tracking fixation-by-fixation, and determined which AOI a child was fixating on using scene analogy stimuli with AOIs overlaid, like the example below, as a reference for what counted as a fixation to a particular AOI. For example, if a fixation was located on or within the immediate area surrounding the featural match (B'), it was manually mapped as a featural match fixation. In addition to the AOIs within the scene analogy stimulus, fixations could also be to the area within the scenes but not included in an AOI, to the surrounding environment external to the scenes, to the experimenter, to the instructor's gestures, or to their own gestures.

Example scene analogy stimulus with AOIs overlaid

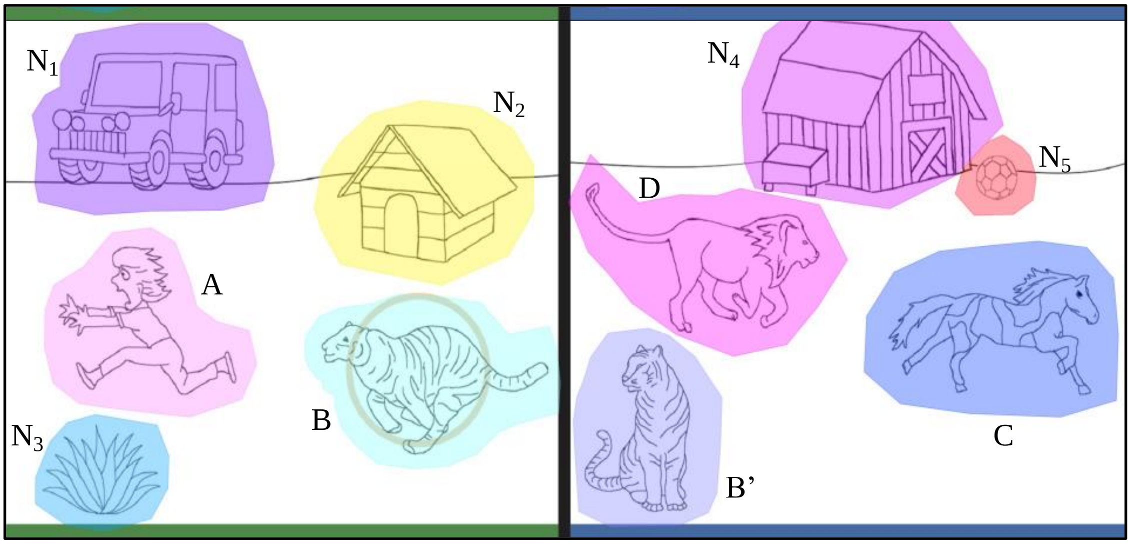

Note: The source scene on the left and the target scene on the right. If item B, the chasing tiger in the source scene, is prompted, the correct choice is item D, the chasing lion in the target scene. Respectively, items B and D and items A and C are in the same part of the pattern (i.e., relationally similar). Item B’ is the featural match that is superficially similar to item B. Items $N_{1\text{-}5}$ are neutral items in the scenes that are not involved in the relation of chasing or the featural match.
